# Burden of Seasonal and Pandemic Influenza-Associated Hospitalization during and after 2009 A(H1N1)pdm09 Pandemic in a Rural Community in India

**DOI:** 10.1371/journal.pone.0055918

**Published:** 2013-05-15

**Authors:** Mandeep S. Chadha, Siddhivinayak Hirve, Fatimah S. Dawood, Pallavi Lele, Avinash Deoshatwar, Somnath Sambhudas, Sanjay Juvekar, Kathryn E. LaFond, Joshua A. Mott, Renu B. Lal, Akhilesh C. Mishra

**Affiliations:** 1 National Institute of Virology, Indian Council of Medical Research, Pune, India; 2 Vadu Rural Health Program, King Edward Memorial Hospital Research Center, Pune, India; 3 Influenza Division, National Center for Immunization and Respiratory Disease, Centers for Disease Control and Prevention, Atlanta, Georgia, United States of America; University of Hong Kong, Hong Kong

## Abstract

**Background:**

Influenza is vaccine-preventable; however, the burden of severe influenza in India remains unknown. We conducted a population-based study to estimate the incidence of laboratory confirmed influenza-associated hospitalizations in a rural community in western India.

**Methods:**

We conducted active surveillance for hospitalized patients with acute medical illnesses or acute chronic disease exacerbations in Pune during pandemic and post pandemic periods (May 2009–April 2011). Nasal and throat swabs were tested for influenza viruses. A community health utilization survey estimated the proportion of residents hospitalized with respiratory illness at non-study facilities and was used to adjust incidence estimates from facility-based surveillance.

**Results:**

Among 9,426 hospitalizations, 3,391 (36%) patients were enrolled; 665 of 3,179 (20.9%) tested positive for influenza. Of 665 influenza positives, 340 (51%) were pandemic A(H1N1)pdm09 and 327 (49%) were seasonal, including A/H3 (16%), A/H1 (3%) and influenza B (30%). The proportion of patients with influenza peaked during August 2009 (39%) and 2010 (42%). The adjusted annual incidence of influenza hospitalizations was 46.8/10,000 during pandemic and 40.5/10,000 during post-pandemic period with comparable incidence of A(H1N1)pdm09 during both periods (18.8 and 20.3, respectively). The incidence of both pH1N1 and seasonal hospitalized influenza disease was highest in the 5–29 year olds.

**Conclusions:**

We document the previously unrecognized burden of influenza hospitalization in a rural community following the emergence of influenza A(H1N1)pdm09 viruses in India. During peak periods of influenza activity circulation i.e during the monsoon period, 20% of all hospital admissions in the community had influenza positivity. These findings can inform development of influenza prevention and control strategies in India.

## Introduction

Though virologic surveillance for human influenza is well established globally [1], limited epidemiologic studies have been carried out in tropical and sub-tropical developing countries [2] and few estimates of influenza disease burden exist for these countries. Excess mortality due to pneumonia and influenza has been used to identify and measure the impact of influenza epidemics in developed countries [3], [4]. Estimates of the incidence of severe non-fatal influenza or influenza-associated hospitalization are another critical measure of influenza disease burden useful for influenza prevention and treatment strategies.

Globally, influenza is a common in children with respiratory infection resulting in substantial burden on the health care system [5]. In India, acute respiratory infections (ARI) are a leading cause of morbidity and mortality, particularly in children less than five years of age in whom the incidence of hospitalized pneumonia is estimated to be 0.37 episodes per child year [5]. An estimated 43 million episodes of ARI occur in India annually. Although several studies have documented that 4–12% of respiratory illnesses in the community are due to influenza [6]–[9] the contribution of influenza to acute medical hospitalizations remains unknown.

Data on severe influenza, such as influenza-associated hospitalization is critical to measuring public health impact of influenza. Understanding this burden in rural communities is particularly important in India where 69% of the population lives in rural areas [10]. Reliable disease burden estimates will assist health care planners in prioritizing investments in health, and evaluating intervention strategies. We conducted a population-based surveillance for patients hospitalized with acute medical illness in a rural community in India to estimate the annual cumulative incidence of influenza-associated hospitalizations during a two year period coinciding with the emergence of the 2009 pandemic. Our data highlights the impact of the 2009 H1N1 pandemic and seasonal influenza and provides the first estimates of influenza-associated hospitalization rates in India.

## Results

### Influenza surveillance among hospitalized patients

From May 2009 to April 2011, 9,426 patients who were hospitalized in the participating hospitals were screened; 3,391 (36%) met eligibility criteria A total of 212 (6%) patients were excluded due to specimen quality, of the remaining 3,179 patients, influenza virus infection was confirmed in 665 (21%) (Table 1),with highest positivity (52%) among persons 15–29 years of age.

**Table 1:** Characteristics of the study population and influenza positivity, by age group, Vadu, District Pune, India, May 2009–April 2011.

	Under surveillance		Enrolled		Influenza positive	
Age, years	No.	%	No.	%	No.	%
*<1r*	1,281	1	86	3	5	1
*1–4*	9,307	8	251	8	39	6
*5–14*	19,262	16	404	13	143	22
*15–29*	43,868	38	1426	45	348	52
*30–44*	24,816	21	554	17	83	12
*45–59*	10,921	9	279	9	33	5
*60+*	7,434	6	179	6	14	2
All ages	116,889	100	3179		665	

The proportion of influenza positive hospitalized patients with acute medical illness was significantly higher during year 1 than year 2 (330/1435 vs. 331/1744 patients, p = 0.007). Pandemic A(H1N1)pdm09 virus predominantly circulated in both years (46% in year 1 and 56% in year 2), followed by influenza B (19% and 40% in year 1 and 2, respectively) and Influenza A (6% A(H1N1) and 29% A(H3N2) in year 1 and 12% A(H3N2) in year 2).

**Figure 1:**
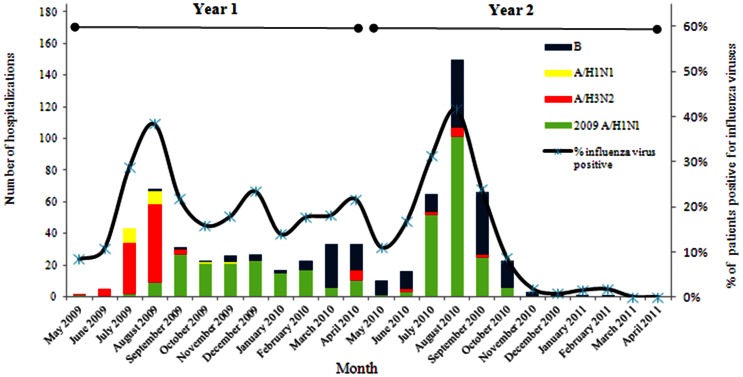
Laboratory confirmed influenza-associated hospitalizations by month of disease onset and influenza type/sub-type, Vadu, District Pune, India, May 2009–April 2011 (n = 665).

The most common diagnosis at discharge was Viral fever (77%), enteric fever (6%), malaria (7%), pneumonia (2%), dengue fever (1.5%) and fever of unknown origin (6.5%),Of the 665 inpatients positive for influenza, 10 (1.5%) had known underlying chronic illness; 8 had chronic obstuctive pulmonary disease/asthma and 2 had CVD. Influenza was associated with ≥10% of hospitalizations for acute medical illness each month during May2009 through September2010 (Figure 1).

The number of influenza-associated hospitalizations peaked in the monsoon season during August of each year. Although pandemic A(H1N1)pdm09 virus was detected in May 2009, the overall monthly peak in influenza-associated hospitalizations during year 1 was associated with A(H3N2) but was lower than the monthly peak of A(H1N1)pdm09-associated hospitalizations during year 2.

### Incidence of all-cause, respiratory, and influenza-associated hospitalizations

Acute medical hospitalizations and respiratory illness hospitalizations were comparable during year 1 and 2 (acute medical hospitalization: 223.6 vs. 229.1 per 10,000 persons; respiratory illness hospitalizations: 149.1 vs. 160.9 per 10,000 persons) (Table 2). Children <5 years had the highest incidences of all-cause acute medical illness and respiratory illness hospitalizations during both years. Duration of hospitalization was upto two days 33.6% patients,3–7 days in 62.6% and more than 7days in 3.7% patients. During year 1 and 2, influenza-associated hospitalizations accounted for 21% and 18% of all acute medical hospitalizations and 25% and 31% of all respiratory hospitalizations, respectively. Among children 5–14 years, influenza-associated hospitalizations accounted for 30–35% of all acute medical hospitalizations and 38–48% of all respiratory hospitalizations.

**Table 2:** Adjusted annual cumulative incidence† per 10,000 persons by age, Vadu, District Pune, India, May 2009–April 2011.

	May 2009–April 2010 (Year 1)*													
	Acute Medical				Respiratory***				Influenza Virus Positive				% of Acute Medical and Respiratory Hospitalizations Associated with Influenza	
Age (years)**	Adjusted Incidence†	95% CI			Adjusted Incidence†	95% CI			Adjusted Incidence†	95% CI			% of acute medical	% of respiratory
<1	463.2	374.3	-	567.9	361.5	281.0	-	453.1	11.3	1.3	-	37.6	2%	3%
1–4	345.5	306.2	-	387.1	244.3	211.8	-	280.6	54.3	39.7	-	73.2	16%	22%
5–14	178.7	159.6	-	199.6	128.7	112.4	-	146.5	61.8	50.6	-	74.5	35%	48%
15–29	268.4	251.6	-	286.2	174.4	160.8	-	188.9	67.1	58.8	-	76.4	25%	38%
30–44	164.4	147.7	-	182.7	100.8	87.6	-	115.2	25.2	19.0	-	33.2	15%	25%
45–59	165.0	140.2	-	192.3	110.0	89.6	-	132.4	18.3	11.1	-	29.7	11%	17%
60+	160.3	130.6	-	193.8	102.2	78.7	-	129.7	11.6	4.4	-	22.6	7%	11%
all ages	223.6	214.4	-	233.0	149.1	141.7	-	156.9	46.8	42.6	-	51.2	21%	31%

	May 2010–April 2011 (Year 2)													
	Acute Medical				Respiratory***				Influenza Virus Positive				% of Acute Medical and Respiratory Hospitalizations Associated with Influenza	
Age (years)**	Adjusted Incidence†	95% CI			Adjusted Incidence†	95% CI			Adjusted Incidence†	95% CI			% of acute medical	% of respiratory
<1	578.1	480.2	-	692.9	455.8	368.3	-	559.0	44.5	21.1	-	87.5	8%	10%
1–4	278.3	246.1	-	314.4	187.7	160.9	-	217.5	36.7	25.6	-	51.5	13%	20%
5–14	126.4	111.1	-	142.9	100.3	86.7	-	115.1	38.4	30.2	-	48.0	30%	38%
15–29	270.3	255.1	-	286.1	190.1	177.2	-	203.4	58.3	51.3	-	66.0	22%	31%
30–44	179.0	162.5	-	196.3	124.2	110.7	-	139.1	23.3	17.5	-	30.0	13%	19%
45–59	249.9	221.5	-	281.7	156.5	133.9	-	182.0	28.8	19.8	-	41.2	12%	18%
60+	244.6	210.2	-	283.3	167.9	139.4	-	200.4	18.7	9.7	-	31.1	8%	11%
all ages	229.1	220.5	-	237.9	160.9	153.7	-	168.3	40.5	36.9	-	44.3	18%	25%

† Incidences were adjusted for health utilization at non-study facilities using the following formula: adjusted hospitalization incidence = (unadjusted incidence)/(% of acute medical hospitalizations reported on health utilization survey that occurred at study facilities).

* From 2009 and 2010 mid-year population estimates from Vadu HDSS.

** Adjustment factor = proportion of acute medical hospitalizations reported on HUS that occurred at study facilities: 0.46 for persons aged <1 year, 0.50 for 1–4 years, 0.78 for 5–14 years, 0.73 for 15–29 years, 0.76 for 30–44 years, 0.68 for 45–59 years, 0.67 for >60 years.

*** Defined as one or more respiratory symptoms in persons ≥5 years: cough, runny nose, sore throat, breathing difficulty or ear ache.

Defined as one or more respiratory symptoms in persons <5 years: cough, runny nose, fast breathing, or ear ache.

**Figure 2:**
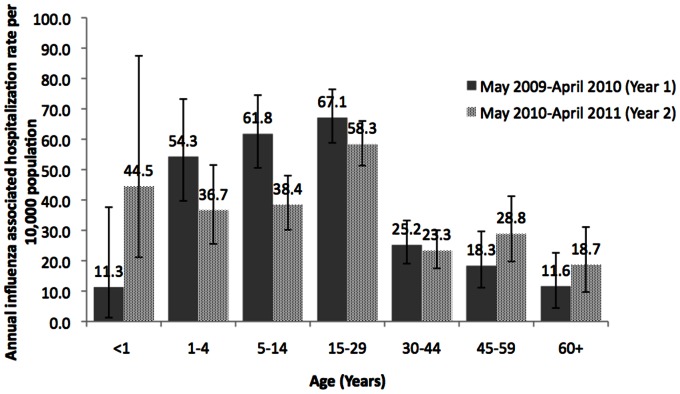
Adjusted annual cumulative incidence of influenza associated hospitalization per 10,000 population, by age, Vadu, District Pune, India, May 2009–April 2011.

Influenza-associated hospitalization was significantly higher (46.8; 95% CI, 42.6–51.2 per10,000 persons) in year 1 than year 2 (40.5; 95% CI, 36.9–44.3 per 10,000 persons) (Table 2). During both years, rates were highest among persons aged 15–29, followed by children aged 5–14 years, and lowest among <1 year olds and persons >60 years (Figure 2).

### Influenza-associated hospitalization incidence by type and sub-type

Influenza A(H1N1)pdm09-associated hospitalization rates were highest (21.5/10,000 persons), followed by seasonal influenza A (16.4/10,000 persons) and influenza B (8.8/10,000 persons) in year 1 (Table 3). During year 2, influenza A(H1N1)pdm09-associated hospitalization rates remained highest (23/10,000 persons), followed by influenza B (16/10,000 persons). Compared with year 1, influenza B-associated hospitalization rates during year 2 were almost two-fold higher (8.8 vs. 16.4/10,000 persons) and seasonal influenza A hospitalization rates were >10 fold lower (16.4 vs. 1.5 per 10,000 persons). Among infants aged <1 year, influenza-associated hospitalizations were exclusively due to influenza B during year 1 and A(H1N1)pdm09 during year 2. In the age groups (1–4, 5–14, 15–29 and 30–44 years)a drop in incidence of influenza A and rise in type B in year2 was observed. In the older age groups of 45–49 and 60 years and above also a fall in influenza A and rise in influenza B as well in influenza A(H1N1)pdm09.

**Table 3:** Adjusted annual cumulative incidence^1^ of influenza associated hospitalization per 10,000 population by age, influenza type and subtype, Vadu, District Pune, India, May 2009–April 2011.

	Year 1: May 2009–April 2010												
		Seasonal A				H1N1pdm09				B			
Age (years)	Adjustment Factor^2^	Adjusted Incidence	(95% CI)			Adjusted Incidence	(95% CI)			Adjusted Incidence	(95% CI)		
<1	0.46	0.0	0.0	-	19.2	0.0	—	-	—	11.3	1.3	-	37.6
1–4	0.50	14.8	7.7	-	26.0	29.6	19.1	-	44.3	9.9	4.3	-	19.6
5–14	0.78	19.1	13.1	-	26.7	30.1	22.4	-	39.3	12.5	8.0	-	19.2
15–29	0.73	21.3	16.7	-	26.8	31.7	26.2	-	38.4	14.1	10.3	-	18.6
30–44	0.76	13.9	9.3	-	20.0	9.4	5.9	-	14.8	1.9	0.5	-	4.9
45–59	0.68	10.7	5.0	-	19.2	6.1	2.3	-	13.6	1.5	0.0	-	5.8
60+	0.67	7.0	1.7	-	16.0	2.3	0.0	-	8.7	2.3	0.0	-	8.7
all ages		16.4	14.0	-	19.2	21.5	18.8	-	24.7	8.8	7.1	-	10.9

	Year 2: May 2010–April 2011												
<1	0.46	0.0	0.0	-	18.9	44.5	21.1	-	87.5	0.0	—	-	—
1–4	0.50	4.3	1.2	-	11.1	21.6	13.3	-	33.5	10.8	5.2	-	19.9
5–14	0.78	0.0	0.0	-	1.9	18.2	12.9	-	25.5	20.2	14.6	-	27.8
15–29	0.73	1.6	0.7	-	3.4	32.5	27.4	-	38.4	24.2	19.7	-	29.3
30–44	0.76	1.6	0.5	-	4.2	14.6	10.1	-	20.1	7.1	4.1	-	11.2
45–59	0.68	2.7	0.6	-	8.2	17.9	10.7	-	27.8	8.2	3.9	-	16.0
60+	0.67	0.0	0.0	-	5.2	10.4	3.9	-	20.2	8.3	3.1	-	18.3
all ages		1.5	0.9	-	2.5	23.0	20.3	-	25.9	16.0	13.7	-	18.4

1 Adjusted hospitalization incidence = (unadjusted incidence)/(% of acute medical hospitalizations reported on health utilization survey that occurred at study facilities).

2 Adjustment factor = proportion of acute medical hospitalizations reported on HUS that occurred at study facilities.

### Estimates of influenza-associated hospitalization incidence by enrollment screening definition

The estimated average annual influenza-associated hospitalization incidence during the study period was 44.1/10,000 persons. Using ILI screening definition for enrollment, the estimated influenza-associated hospitalization incidence is 30.1/10,000 persons, (68% of the burden estimate resulting from enrolling patients with all acute medical illness) (Table 4) and using SARI screening definition it is 1.3/10,000 persons which are significant underestimates.

**Table 4:** Effect of screening case definitions on estimates of influenza-associated hospitalization annual incidence, Vadu, District Pune, India, May 2009–April 2011.

Screending definition	# of patients meeting screening definition	# (%) patients with specimens positive for influenza virus	Adjusted influenza-associated hospitalization annual incidence per 10,000 persons	% underestimate*
Acute medical hospitalization	3179	665 (20,9%)	44.1	—
ILI**	1632	454 (27.8%)	30.1	32%
ARI***	2183	566 (25.9%)	37.5	15%
FARI†	1659	455 (27.4%)	30.1	32%
SARI‡	87	20 (23.0%)	1.3	97%

* % underestimate defined as ((1-estimated hospitalization incidence based on use of the given screening definition)/estimated hospitalization incidence based on use of all acute medical hospitalizations as a screening definition) expressed as a percentage).

** Influenza-like illness (ILI) was defined as fever and either cough or sore throat.

*** Acute respiratory illness (ARI) was defined as >1 of the following: cough, nasal discharge, sore throat, or shortness of breath (based on history).

† Febrile acute respiratory illness (FARI) was defined as the presence of ARI plus fever.

‡ For persons >5 years, severe acute respiratory illness (SARI) was defined as the presence of ILI and either shortness of breath or difficulty breathing.

For children <5 years, SARI was defined as pneumonia or severe pneumonia as defined by the Integrated Management of Childhood Illness guidelines as any of the following: any general danger sign (i.e. unable to drink or breastfeed, vomiting, convulsions, lethargic, or unconscious), chest indrawing, stridor, or fast breathing on examination.

## Discussion

Our study is the first attempt to estimate burden of influenza among acute medical hospitalized patients in a large population based study in rural India. Adopting a broad surveillance case definition ensured that influenza cases presenting as acute exacerbations of chronic disease or having atypical presentations were not missed. However, the numbers of patients with underlying chronic disease is low; there is a possibility that chronic diseases go undiagnosed in the community. Population-based surveillance in a well-enumerated population and health utilization surveys enabling adjustment for patients admitted to non-participating hospitals allowed us to better estimate the annual incidence of all acute medical illness hospitalizations, respiratory hospitalizations, as well as influenza-associated hospitalizations. I Influenza accounted for a substantial proportion of all acute medical and respiratory illness hospitalizations during and after the emergence of the A(H1N1)pdm09 virus. Use of ILI or SARI case definitions, to estimate the burden of influenza-associated hospitalizations would have substantially underestimated the impact of influenza in the study population.

The average annualized incidence of influenza-associated hospitalizations in current study was 44.1 per 10,000 persons, which is substantially higher than 3.6–11.5/10,000 persons reported in the United States [11]. We undertook this burden study when a pandemic virus was emerging, thus our estimated incidence rates of influenza-associated hospitalizations are higher than what has been observed for seasonal influenza elsewhere. We found the highest influenza-associated hospitalization incidences occurred among persons aged 5–29 years which differs markedly from findings for seasonal influenza in other countries. In the US and Canada, influenza-associated hospitalization incidences are highest among those >65 years with underlying medical conditions (40–56 per 10,000), infants aged <6 months (18–104 per 10,000) and adults >65 years without underlying conditions (9–23 per 10,000) [12]–[18]. Similarly, in Thailand and Hong Kong, hospitalization incidences are highest among older adults, and incidences among children exceed those in the United States [19]–[23].In contrast, we found the lowest hospitalization incidences in persons aged >60 years, followed by infants aged <1 year. The difference in the relative age distribution of influenza-associated hospitalization incidence between our study and others might be due to the A(H1N1)pdm09 virus which resulted in increased morbidity and mortality among younger persons compared to seasonal influenza. However, persons aged 1–29 years had the highest hospitalization incidence for acute medical illness, respiratory illness, and influenza suggesting that our population is likely to have differed from populations in other studies in propensity to seek care. Despite these differences, the incidence estimates for infants aged <1 year and children aged 1 to 5 years in our study are similar to the estimates from the US for similar age groups [24],[25], suggesting that these age groups be targeted for influenza prevention strategies.

In our population, 46–57% of the burden of influenza-associated hospitalizations was accounted for by A(H1N1)pdm09 influenza virus with seasonal influenza A virus accounting for 35% of the burden in 2009–10 and 4% in 2010–11. Children and young adults bore a disproportionate burden of A(H1N1)pdm09-associated hospitalizations, possibly due to lack of prior exposure to similar viruses. In a study carried out in Pune, high rates of influenza-associated hospitalizations and deaths among persons aged <35 were observed during the peak of A(H1N1)pdm09 activity in August-September 2009 [26]. Influenza B virus infection accounted for 19–39% of all influenza-associated hospitalizations in our study, despite conceptions that influenza B is typically associated with milder disease [27].

The seasonality of influenza virus circulation and seasonal patterns for excess hospitalizations for pneumonia and influenza during the winter are known for temperate countries [28]. Some tropical and sub-tropical countries have documented significant transmission throughout the year while others have documented a biannual pattern [28]. Although the A(H1N1)pdm09 virus resulted in influenza-associated hospitalizations continuously during July2009–April2011, we documented a clear seasonality to influenza-associated hospitalizations in our study with peaks during the monsoon season each year. In year 1, Influenza A(H3N2) predominantly contributed to the peak and A(H1N1)pdm09 in year 2. A previous multisite study conducted from 2004 to 2008 identified similar seasonal patterns for influenza in western India, similarly, influenza activity has been shown to peak in the monsoon season in other parts of India [6],[7].

Our study has certain limitations. 6% patients enrolled were not included in the study due to poor quality of samples collected and approximately 10% patients were sampled more than seven days after onset of illness when virus shedding may have ceased by then, this could have led to missing some cases.

At the onset of this study to assess the burden of seasonal influenza-associated hospitalizations, the A(H1N1)pdm09 virus emerged. Thus, our estimates may not reflect influenza-associated hospitalization burden during typical seasonal influenza epidemics. Nevertheless, our study does document both seasonal and A(H1N1)pdm09-associated hospitalization burden during the 2 years following emergence of the A(H1N1)pdm09 virus. The study is ongoing and will produce estimates of influenza-associated hospitalization for more typical seasonal influenza epidemics in the future. Rates of hospitalization were high in comparison to other countries, it is possible that the media publicity, increased public awareness, and panic amongst the public following deaths due to the H1N1pdm influenza virus in 2009 could have unusually altered population's propensity to seek care so also heightened provider awareness and responsiveness to the pandemic. However, the rate of hospitalization continued to be high even in the 2^nd^ year of our study when public and provider panic had subsided.

While we could have over-estimated the influenza burden in first year of study, we observed comparable rates of influenza burden in second year. India is a large country with diverse geography, demography, and climate, and our estimates of influenza-associated hospitalization do not necessarily reflect the impact of influenza in all regions of the country. However, the study area is representative of a phenomenon that is increasingly seen across India viz. fast-growing communities situated not far from urban area.

Rates of hospitalization, especially related to influenza-associated illness, are critical to measuring public health impact of influenza in order to enable countries to make informed evidence-based decisions while allocating scarce resources towards prevention and control. Reliable disease burden estimates will assist health care planners in prioritizing investments in health and research, improving access to health care and evaluating intervention strategies.

## Methods

### Study area and population characteristics

A rural population in 22 villages within a geographical area of 232 sq. km in Pune District, India has been under household demographic surveillance (HDS) since 2001. A bi-annual census enumerates birth, migration, and death events and ascertains cause of death through verbal autopsy. In May 2010, the study site included a population of 116,898 persons comprising 34,181 households. A health utilization survey was conducted six-monthly to enumerate hospitalizations and determine cause and place of hospitalization (within/outside the study area). The proportion of ARI-associated hospitalizations occurring outside the area was estimated for the population under demographic surveillance and used to adjust the incidence of influenza-associated hospitalization for the study area.

The study area Vadu, is located 30 km north-east of Pune City in Western India and has a health structure typical of a rural area in close proximity of a large city. The public sector services include a rural hospital and primary health centers located in villages. The private sector several small general hospitals where most residents seek health care. The study population is served by 30 hospitals (a 35-bed rural hospital at Vadu, a 5-bed primary health center, and 28 private nursing homes having 2–30 (median 10) beds each). The enrolled hospitals are small private hospitals which provide medical care for patients through their out-patient clinics and are not necessarily referral hospitals wherein serious patients are referred by health care providers. The majority of admissions to these hospitals occur for patients who seek medical care in their outpatient clinic and are then advised admission. Unfortunately we did not record whether the patient came directly to the hospital in an emergency or was admitted after an outpatient consultation.

The area is well connected with Pune, a city, 30 kilometers away. Winters (November–March) are mild, and the monsoon season from June through September.

### Surveillance for influenza-associated hospitalization

Hospital-based surveillance was initiated in May, 2009 in 29 of 30 hospitals (One private hospital did not consent to participate). Field-based investigators screened all overnight hospital admissions daily. Using a broad case definition, all patients presenting to a hospital with a medical illness with acute onset of respiratory symptoms, fever or history of fever within the past week, or acute exacerbation of a pre-existing chronic medical condition (chronic lung disease, asthma, cardio-vascular disease) were enrolled. Patients residing outside HDS area, and patients hospitalized for elective/emergency surgeries, trauma, orthopedic, ophthalmological, psychiatric or obstetric care were excluded. Data on clinical symptoms, signs, and treatment were collected on admission, at time of interview and at discharge including patient outcome. Written informed consent was obtained from each study participant (or parent/legal guardian for persons <18 years) prior to enrolment. The study protocol was reviewed and approved by the Institutional Review Boards of the National Institute of Virology (NIV), Pune, King Edward Memorial Hospital, and the USCDC, Atlanta.

### Laboratory testing

Nasal and/or throat swabs were collected and combined into a single vial of virus transport medium [29] and transported at 2–8C to the NI V, Pune for virologic testing. Real time RT-PCR was used to detect influenza viruses using the CDC protocol [30].

### Data analysis

Year one was May2009–April2010 and year two May2010–Apri2011. Annual cumulative incidence of influenza-associated hospitalizations unadjusted for healthcare utilization outside the surveillance area was the number of laboratory-confirmed influenza-associated hospitalizations per year divided by 2009 and 2010 population. The annual cumulative incidence adjusted for healthcare utilization patterns was calculated by dividing the unadjusted annual cumulative incidence by the proportion of acute respiratory illness hospitalizations among HDS area residents recorded at facilities under surveillance.

Unadjusted and adjusted cumulative incidences of acute-medical illness, all-cause respiratory illness and influenza-associated hospitalization were calculated by influenza type and subtype and by age group.

To evaluate the effect of screening definitions on the estimate of influenza-associated hospitalization incidence, incidence was also calculated using patients with laboratory-confirmed influenza who met commonly used screening definitions for influenza divided by population denominators. These included influenza-like illness (ILI) defined as measured fever >38^0^ and cough or sore throat; severe acute respiratory illness (SARI) defined as ILI plus shortness of breath or difficulty in breathing for patients >5 years of age OR the WHO IMCI definition of pneumonia for those <5 years of age; acute respiratory illness (ARI) defined as sudden onset and ≥1 of the respiratory symptoms: cough, sore throat, shortness of breath, or nasal discharge; and febrile acute respiratory illness (FARI) defined as ARI plus measured fever >38^0^.
